# Case report: a two-step approach in the management of angle-closure glaucoma associated with Plateau iris configuration

**DOI:** 10.5935/0004-2749.2025-0020

**Published:** 2025-08-12

**Authors:** Tiago Santos Prata, Isabella Cristina Tristão Pinto Resende, Daniela Mauricio Ribeiro, Fábio Nishimura Kanadani, Izabela Negrão Frota de Almeida

**Affiliations:** 1 Department of Ophthalmology and Visual Science, Escola Paulista de Medicina, Universidade Federal de São Paulo, São Paulo, SP, Brazil; 2 Glaucoma Institute, São Paulo, SP, Brazil; 3 Department of Ophthalmology, Mayo Clinic, Jacksonville, Florida, United States; 4 Department of Medicine, Universidade de Itaúna, Itaúna, MG, Brazil; 5 Department of Ophthalmology, Universidade Federal do Pará, Belém, PA, Brazil

**Keywords:** Glaucoma, angle-closure/surgery, Iris diseases/surgery, Laser coagulation/methods, Phacoemulsification, Lens implantation, intraocular, Case reports

## Abstract

Angle-closure glaucoma is a major cause of visual impairment worldwide, with Plateau iris syndrome presenting management challenges. We present a case report of a 58-year-old woman with advanced, uncontrolled angle--closure glaucoma and Plateau iris. Her history included laser peripheral iridotomy and three glaucoma medications in both eyes. Different treatments were implemented. For the eye with lower intraocular pressure, fewer peripheral anterior synechiae, and milder disease: phacoemulsification with intraocular lens implantation. For the eye with more advanced disease, a two-step approach was used: slow-coagulation transscleral cyclophotocoagulation using the double-arc protocol, followed by phacoemulsification with intraocular lens implantation 2 months later. Both eyes achieved improved visual acuity and intraocular pressure control with fewer medications, without significant complications. This case highlights transscleral cyclophotocoagulation followed by phacoemulsification as an alternative to combined surgeries in uncontrolled angle-closure glaucoma with Plateau iris, offering a simpler technique, more predictable refractive and pressure-control outcomes, and more straightforward postoperative management.

## INTRODUCTION

Angle-closure glaucoma (ACG) is a major cause of visual impairment worldwide. It is characterized by elevated intraocular pressure (IOP) due to impaired drainage of aqueous humor, often resulting from anatomic abnormalities that block the trabecular meshwork. Plateau iris syndrome presents unique management challenges among the forms of ACG due to its distinct anatomical configuration^(1)^.

Regarding treatment, laser peripheral iridotomy (LPI), the first-line therapy, is insufficient if there is persistent IOP elevation or extensive peripheral anterior synechiae (PAS)^(1)^. Phacoemulsification also deepens the anterior chamber (AC) and widens the angle, relieving IOP^(2)^. However, in more advanced disease or anatomical challenges, it may not adequately control IOP, requiring combined or sequential interventions^(3)^.

Traditionally, combined phacoemulsification and trabeculectomy are used in advanced cases but carry a higher risk of complications, particularly in eyes with Plateau iris^(3)^. An alternative is a two-step strategy using transscleral cyclophotocoagulation (TSCPC) with the double-arc slow-coagulation TSCPC protocol (DA-TSCPC), followed by phacoemulsification. This approach may be safe and effective for complex cases^(4)^.

A PubMed literature review on September 16, 2024, using the keywords “Transscleral Cyclophotocoagulation” and “Plateau iris”, identified one successful case of ACG and Plateau iris treated with combined phacoe-mulsification, goniotomy, and TSCPC^(5)^. No reports of DA-TSCPC followed by phacoemulsification were found. This case presents a strategy for managing uncontrolled ACG and Plateau iris configuration, offering insights into the benefits and considerations of a staged surgical approach.

## CASE REPORT

A healthy 58-year-old White female was referred due to uncontrolled ACG, on three glaucoma medications (brinzolamide plus timolol maleate, and latanoprost in both eyes - OU), treated for 9 years. Her ocular history included only laser peripheral iridotomy (LPI) in OU.

**Figure 1:**
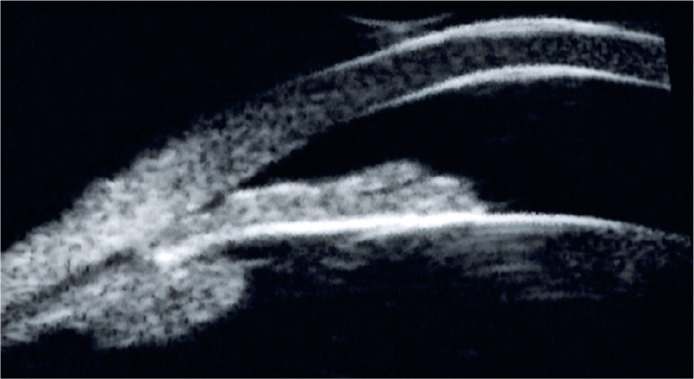
Ultrasound biomicroscopy (UBM) shows anteriorly positioned ciliary processes, a narrow or absent ciliary sulcus, and a flat central iris with steep peripheral angulation, consistent with a Plateau iris configuration.

Best-corrected visual acuity (BCVA) was 20/25 in OU, with mild hyperopia (+1.50 -0.75 **×** 135 in the right eye - OD; +1.25 -0.50 **×** 160 in the left eye - OS). IOP was 25 mmHg in OD and 17 mmHg in OS. Biomicroscopy revealed mild cataracts, a shallow peripheral anterior chamber (AC), and a relatively deep central AC in OU. Gonioscopy showed occludable angles with the double hump sign during indentation, extensive peripheral anterior synechiae (PAS) in OD, and appositional angle closure with localized PAS in OS. Fundus examination showed enlarged disc cupping, more pronounced in OD (cup-to-disc ratio of 0.85 OD and 0.7 OS), with neuroretinal rim thinning confirmed by structural and functional tests (Mean Deviation: -11.82 and -4.11, OD and OS, respectively). Ultrasound biomicroscopy revealed a Plateau iris configuration in OU (Figure 1).

Given the findings, different approaches were used for each eye. For OS, which had lower IOP and fewer PAS, phacoemulsification with intraocular lens (IOL) implantation was performed. For OD, which had higher IOP, extensive PAS, and more advanced disease, a two--step approach was used: DA-TSCPC^(4)^ followed by phacoemulsification with IOL implantation 2 months later.

**Figure 2:**
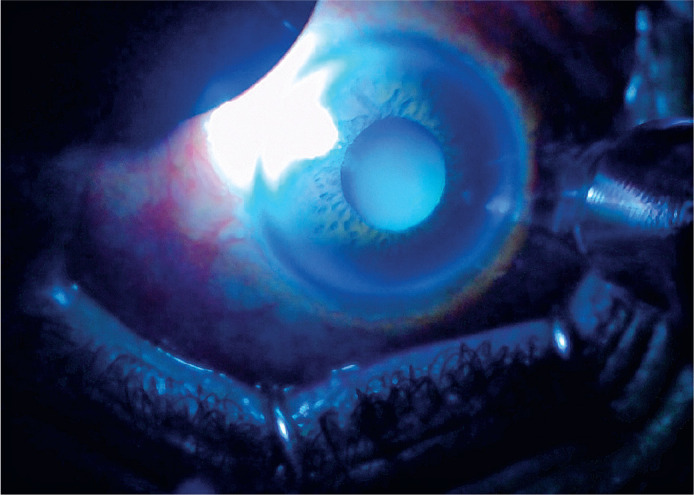
The ciliary body position was determined by transillumination.

**Figure 3:**
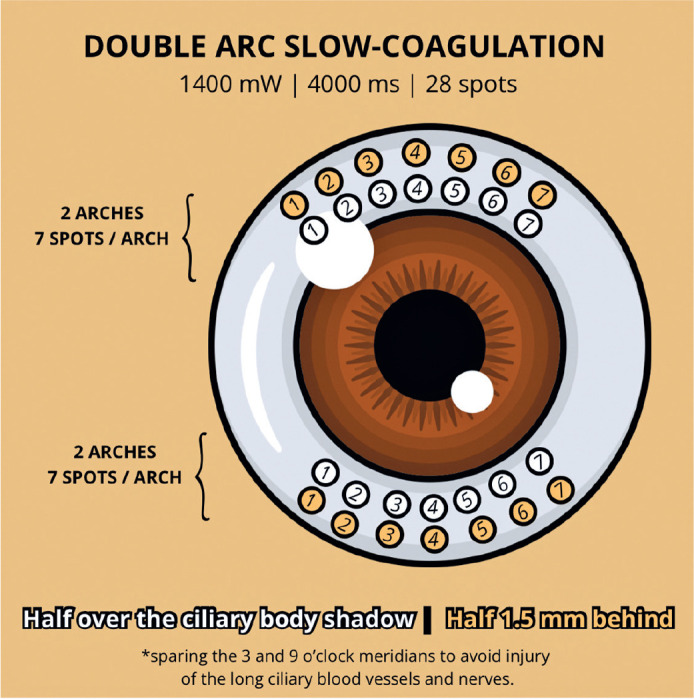
The double-arc slow-coagulation transscleral cyclophotocoagulation technique has 28 applications divided into four rows: two superior and two inferior (upper and lower arcs).

DA-TSCPC was performed under peribulbar anesthesia using a transillumination microscope (Figure 2) and a conventional 810 nm diode laser with a laser probe (Lightmed Corporation, San Clemente, CA, USA). The settings were 1400 mW and 4000 ms. A total of 28 applications were applied in four rows: upper and lower arcs (Figure 3). For each arc, 7 spots were placed over the ciliary body shadow (perilimbal dark band) and 7 spots 1.5 mm behind, sparing the 3 and 9 o’clock meridians. No “POP sound” indicative of ciliary body explosion was heard. The patient was prescribed 0.1% nepafenac 4 times/day for 15 days and 1% prednisolone acetate 4 times/day, tapered by removing one dose each week.

Six weeks after surgery in OS, IOP was 14 mmHg with two topical medications. Six weeks after DA-TSCPC in OD, IOP was 13 mmHg with three topical medications. Following phacoemulsification in OD, one medication was discontinued. After six months, BCVA was 20/20 in OU (refraction: +0.50 -0.50 **×** 145 and +0.25 -0.50 **×** 150, OD and OS, respectively), and medications remained unchanged (brinzolamide plus timolol maleate in OU), with IOP at 14 and 12 mmHg. No significant postoperative pupillary diameter changes were observed in OU.

## DISCUSSION

This case highlights the complexity of managing ACG, particularly in eyes with Plateau iris. Although many cases can be managed with phacoemulsification^(2)^, eyes with uncontrolled IOP and advanced damage often require a combined procedure, such as phacoemulsification and trabeculectomy^(3)^.

However, combined procedures can be challenging, especially in the presence of Plateau iris, a risk factor for postoperative malignant glaucoma^(6)^. This underscores the need to explore surgical techniques that improve patient safety in such cases.

In this case, a two-step approach was used for OD instead of the conventional combined procedure. We believe this strategy offers advantages, particularly in eyes with good visual potential^(4)^. Dividing the treatment simplifies each step, reduces aggressiveness, improves postoperative inflammation control, and potentially lowers the risk of complications associated with combined phacoemulsification and cyclophotocoagulation (CPC). It also allows for more straightforward postoperative management^(7)^.

Additionally, performing CPC before cataract surgery enhances predictability in pressure control, which enables more precise surgical planning^(4)^. It also offers flexibility to repeat CPC or adjust the technique if the initial outcome is suboptimal^(8)^. However, this approach requires two visits to the surgical center under anesthesia.

Diode laser TSCPC has been successfully used to treat chronic ACG, with a one-year success rate over 80%^(9)^. In this case, it eliminated the need for a filtering procedure and reduced the risk of ocular decompression in OD. Evidence also suggests that laser application to the ciliary body may deepen the AC, potentially making phacoemulsification easier and refractive outcomes more predictable^(10)^.

While potential complications of TSCPC include prolonged inflammation and hypotony, our one-year safety profile of DA-TSCPC showed that complications were infrequent and transient, especially in eyes with better visual prognosis^(4)^.

In summary, this case demonstrates the potential of TSCPC as a preparatory step before phacoemulsification in eyes with uncontrolled ACG and Plateau iris. This approach may provide a safer and more effective alternative to traditional combined procedures. The case is part of an ongoing prospective study, with one-year results pending publication. However, studies with larger sample sizes and longer follow-up are needed to confirm the safety and efficacy of this two-step approach.

## Data Availability

The datasets generated and/or analyzed during the current study are included in the manuscript.
